# Selection and quantification of infection endpoints for trials of vaccines against intestinal helminths

**DOI:** 10.1016/j.vaccine.2011.03.026

**Published:** 2011-05-09

**Authors:** Neal Alexander, Bonnie Cundill, Lorenzo Sabatelli, Jeffrey M. Bethony, David Diemert, Peter Hotez, Peter G. Smith, Laura C. Rodrigues, Simon Brooker

**Affiliations:** a London School of Hygiene and Tropical Medicine, London, UK; b The George Washington University, Washington, DC, USA; c Centro de Pesquisas René Rachou (FIOCRUZ), Belo Horizonte, Minas Gerais, Brazil; d Sabin Vaccine Institute, Washington, DC, USA

**Keywords:** Helminth vaccines, Parasitological endpoints, Mathematical model

## Abstract

Vaccines against human helminths are being developed but the choice of optimal parasitological endpoints and effect measures to assess their efficacy has received little attention. Assuming negative binomial distributions for the parasite counts, we rank the statistical power of three measures of efficacy: ratio of mean parasite intensity at the end of the trial, the odds ratio of infection at the end of the trial, and the rate ratio of incidence of infection during the trial. We also use a modelling approach to estimate the likely impact of trial interventions on the force of infection, and hence statistical power. We conclude that (1) final mean parasite intensity is a suitable endpoint for later phase vaccine trials, and (2) mass effects of trial interventions are unlikely to appreciably reduce the force of infection in the community – and hence statistical power – unless there is a combination of high vaccine efficacy and a large proportion of the population enrolled.

## 1 Introduction

Intestinal helminths infect more than one billion people worldwide [1] and cause the loss of more than four million disability-adjusted life years (DALYs) annually [2]. The most important genera are *Ascaris*, *Trichuris*, *Ancylostoma*, *Necator*, *Enterobius* and *Schistosoma* [2,3], while others include *Taenia*, *Hymenolepis* and *Fasciola*. Current control efforts rely more heavily on chemotherapy than on sanitary measures [1,2,4]. However, there are concerns about the sustainability of periodic deworming on the basis of cost, and risk of drug resistance. This has prompted development of vaccines against intestinal parasites of humans [5]. Trials of vaccines against hookworm have reached Phase I [6] while those against and *Schistosoma japonicum* and *S*. *mansoni* are in pre-clinical studies [7–9]. Veterinary vaccines have also been developed against *Fasciola hepatica* [10], *Taenia* spp and *Echinococcus granulosus* [11].

Intestinal helminths generally cause greater morbidity at higher infection intensity [3,4], and the vaccines currently in development are more likely to reduce the intensity of infections than to confer complete protection against infection. For these reasons, trial endpoints for such vaccines are likely to include measures of the intensity of infection as well as just the presence or absence of infection. Such intensity endpoints have not, so far, been commonly used as primary measures of efficacy in vaccine trials. Trials of existing vaccines against non-helminth parasitic diseases, notably malaria, have concentrated on protection against infection considered as a dichotomous variable: either infection of any intensity, or clinical symptoms combined with infection above a specified threshold. In trials in which the primary endpoint is the incidence of new infections, existing infections are usually cleared at the start of the trial, and typical endpoints are the cumulative proportion infected, or the infection incidence rate [12–18]. If infection intensity is analysed as an endpoint in itself, it is usually a secondary endpoint.

An exception is the Combination B malaria vaccine trial in Papua New Guinea [19]. This is instructive because those subjects who were free of parasitaemia throughout follow-up were not included in the analysis, apparently because zeros were not accommodated by the chosen analysis, which used logarithms of geometric mean infection intensity. In the current paper we will use arithmetic rather than geometric means to measure infection intensity. This avoids problems with zeros but, more importantly, the arithmetic mean has a closer link with the force of infection, i.e. the rate of acquisition of infection. The force of infection is likely to be approximately proportional to the total number of infectious parasites in the community. In the case of hookworm, this is the total number of free-living larvae, which in turn is likely to be approximately proportional to the total eggs excreted per unit time, which by definition is proportional to the arithmetic mean egg count. By contrast, the geometric mean has no clear relation with the force of infection.

We will assess the statistical power of assessing efficacy measures based upon the following endpoints: (i) arithmetic mean parasite intensity at the end of the trial, (ii) dichotomous infection status (i.e., whether or not infected) during the trial, and (iii) incidence rate of first infection following vaccination, based on time to first infection. The corresponding efficacy measures will be (i) between-arm ratio of means, (ii) odds ratio and (iii) rate ratio. We will also consider the importance of the reduction in the force of infection in the trial population caused by clearing prevalent infections among trial participants at the start of the trial, and vaccinating some of the population. The work has been motivated by the design of preliminary studies and trials within the Human Hookworm Vaccine Initiative [20].

## 2 Materials and methods

### 2.1 Statistical power of potential efficacy measures

We consider simple two-arm trial designs consisting of equal numbers of individuals randomized between vaccinated and unvaccinated arms. Any product administered to those in the unvaccinated arm is assumed to be ineffective against the target infection. We rank the statistical power of ratio measures of efficacy based on the following three endpoints: (1) mean infection intensity at the end of the trial; (2) whether infected or not at the end of the trial; and (3) incidence rate of first infection in the trial. We assume that infection intensity is estimated by counting parasites in a standard stool sample volume, and that these parasite counts can be described by a negative binomial distribution, as is commonly the case [21,22]. This distribution is a generalization of the Poisson. While the Poisson is mathematically based on an assumption of uniformity in the underlying level of parasites in different individuals, the negative binomial has an additional parameter (usually denoted *k*) which allows greater variance, reflecting between-person heterogeneity in infection levels. We express the parameters of the other two endpoints in terms of those of the negative binomial distribution. For each endpoint we obtain the test statistic for comparing the vaccine and control arms, as the ratio of a relevant efficacy measure divided by its standard error. For mean parasite intensity, the efficacy measure is the ratio of means between the two arms; for dichotomous infection status, it is the odds ratio; and for the incidence of first infection, it is the rate ratio. We further assume that there will be no waning of efficacy during the period of the trial. Appendix A shows the derivation of the corresponding expected values of the test statistics which are denoted *Z*_*mean*_, *Z*_*OR*_ and *Z*_*RR*_. Higher absolute values of these *Z* statistics correspond to greater evidence against the null hypothesis for a given set of trial parameters (such as vaccine efficacy) with assumed values. We treat this as synonymous with greater statistical power, although it is a simpler concept than pre-trial power calculation, which depends on the *Z* statistics under the null and alternative hypotheses [23]. Trial duration does not enter directly into our calculations, although longer trials will tend to have larger infection intensities in the control arm, and hence higher efficacy measures (under the above assumption of no vaccine waning).

The test statistics (*Z*) are expressed in terms of the negative binomial's mean (*μ*) and dispersion (*k*) parameters. The latter is associated with variance *V* via the relation *V* = *μ* + *μ*^2^/*k*, so lower values of *k* imply higher values of variance. For sample size calculations, the simplest assumption is that *k* holds a constant value over the different arms of the trial [24]. However, descriptive studies often find lower values of *μ* to be associated with lower values of *k*. We therefore also allow for this possibility within trials. Since the vaccine is assumed to reduce *μ* from its pre-trial value, this implies a smaller *k* in the vaccine arm, relative to control. So, an assumption of such a relation between *μ* and *k* is conservative, in that it increases the variance, relative to the previous assumption of constant *k*.

To quantify this reduction in *k*, we use Taylor's power law, which is an empirical relation between mean and variance of population numbers: *V* = *aμ*^*b*^ [25,26]. The magnitude of association is reflected by the *b* parameter, while *a* is of less fundamental interest since it depends on sampling effort. Equating this power law with the previous expression for *V* gives *k* = *μ*/(*aμ*^*b*−1^ − 1), or *k* ≈ *μ*^2−*b*^/*a* [27]. Shaw [28] found that *b* was approximately 1.5 for many different parasite taxa, including nematodes. For hookworm, this is consistent with data from our studies conducted in Americaninhas (Minais Gerais State, Brazil) [29], from which we estimated *b* = 1.4 (95% confidence interval 0.6–2.2). Hence we use a value of *b* = 1.5 in our analysis, so that the value *k*_1_ in the trial arm is related to that in the control arm by $k_{1}=k_{0}\sqrt{\mu 1/\mu 0}$.

When calculating the test statistic for incidence (Z_*RR*), we take account of the fact that monitoring of new infections will not generally be continuous, so they will not be detected till the next scheduled follow-up examination. This increases the standard error of the rate ratio to an extent which depends on the frequency of follow-up [30].

### 2.2 Impact on force of infection of trial interventions

The statistical power of a vaccine trial will depend in part on the force of infection in the study area (i.e. rate of acquisition of infection). The greater the force of infection, the greater the expected number of infection events in the trial, and the greater the statistical power of the trial. However, the trial intervention(s), if efficacious, will tend to reduce this force of infection to below the pre-trial value, even in the control arm, because their vaccinated neighbours will, on average, be less infectious. For the present work, we assume that all infections are cleared at the start in both arms of the trial (vaccine and control). We modify an existing model to quantify the impact of these interventions on the time course of the mean number of adult worms per person, in both arms of the trial, and in those people not participating. We assume that:

a proportion *ϕ* of the study area's population are trial participants, half of these in the vaccine arm and half in the control arm;
the vaccine reduces acquisition of adult worms by a factor *ψ*, and this protection is assumed to last at least for the duration of the trial.

For this analysis, we assumed *k* to be lower for lower values of *μ*, via the relation derived above $\left(k_{1}=k_{0}\sqrt{\mu 1/\mu 0}\right)$. The model equations are derived in Appendix B.

All calculations were done using the software S-Plus versions 6.2 and 7, and R versions 2.5–2.7. In particular, the model's differential equations were solved numerically using the ‘odesolve’ package in R.

## 3 Results

### 3.1 Statistical power of potential efficacy measures

**Fig. 1:**
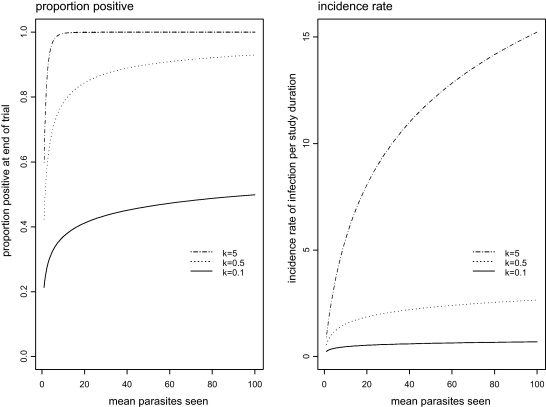
Relation between the proportion parasite-positive at the end of the trial, incidence rate, and the two parameters of the negative binomial distribution: the mean parasite intensity (*μ*), and the *k* dispersion parameter.

We consider three candidate efficacy measures: ratio of mean parasite intensity at the end of the trial, the odds ratio for being parasite-positive at the end of the trial, and the incidence rate ratio for incidence of infection (of any intensity) during the trial. All three are expressed in terms of the parameters of the negative binomial distribution: the mean (*μ*) and dispersion parameter *k* (Appendix A). The mean is of the number of parasites seen per person at each examination round. For example, for intestinal parasites, it could be the mean of the total numbers of eggs actually seen (added up over possibly multiple slides). For example, from a single Kato-Katz slide per person, the possible values of eggs seen are 0, 1, 2, … and the possible values of eggs per gram are 0, 24, 48, …. The negative binomial distribution can be fitted to the former data but not the latter. However, if the resulting mean is, for example, 2 eggs per slide, this is easily converted to 48 eggs per gram. Larger values of *k* parameter imply a distribution of parasites between people which is more uniform (closer to Poisson). Equivalently, *k* is smaller when parasites are more clustered in smaller numbers of people. Hence, for a given mean parasite intensity, larger values of *k* correspond to larger prevalences, and larger incidence rates. This is illustrated in Fig. 1.

**Fig. 2:**
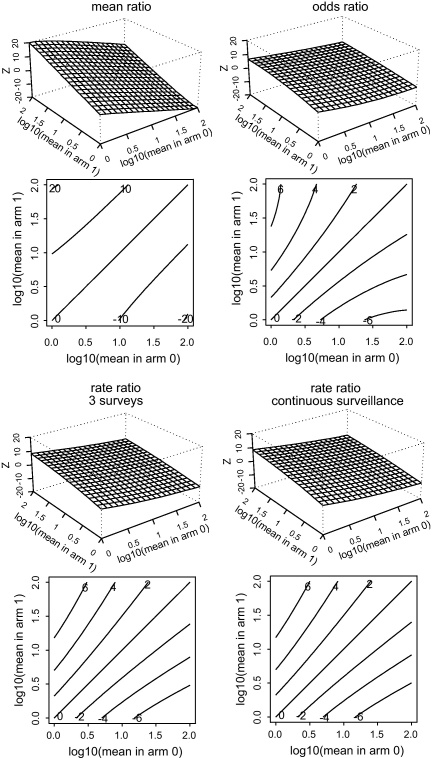
Surface and contour plots of *Z* statistics from four candidate effect measures. The horizontal axes in the surface plots, and both axes in the contour plots, show possible values of mean infection intensity (number of parasites seen per person) in the two arms (‘0’ and ‘1’) of a trial with 100 people per arm and a fixed value of 0.5 for negative binomial dispersion parameter (*k*). The vertical axis in the surface plot, and the contour values in the contour plot, show *Z* statistics: the expected value of the test statistic divided by its standard error. A *Z* statistic of 2 (or −2) corresponds to a two-sided *p* value of 0.05, and *Z* = 4 corresponds to *p* = 0.00006. Comparing the effect measures, a higher *Z* value for the same means indicates greater statistical power. For any combination of mean infection intensity, the ratio of means has the highest absolute value of *Z*, and so is the most powerful effect measure, followed by the rate ratio. The ratio of means achieves *Z* values more than 20, while the others do not reach *Z* = 10. Whether the rate estimation is based on three examination rounds, or continuous surveillance, makes little difference, as indicated by comparing their *Z* values for any given combination of mean infection intensities in the two arms. Finally, the odds ratio is slightly less powerful than the rate ratio. This can be seen, for example, by noting that the latter reaches values of *Z* = ±6 for slightly smaller differences in means: these contours are further in from the upper left and bottom right corners.

To rank the statistical power of the three effect measures, we calculate their *Z* statistics, i.e. the ratio of expected value to standard error. This is illustrated in Box, and in Fig. 2 for a range of mean eggs counted per person (*μ*) in the control and intervention arms, for a fixed *k* of 0.5 and *n* = 100 people per arm. Each of the effect measures has a three-dimensional surface plot and a contour plot, showing the same results in terms of its *Z* statistic. A *Z* value which is larger in magnitude (i.e. farther from zero) produces a smaller *p* value and reflects greater statistical power. Fig. 2 shows that, for the fixed value of *k* = 0.5, the ratio of means is more powerful than the other measures. The next most powerful is the incidence rate ratio, followed by the odds ratio. Little gain in power results from measuring incidence continually as opposed to three follow-up surveys. One would expect the odds ratio to be less powerful than the incidence rate ratio because, while both are based on infection status as a binary outcome, the odds ratio is based only on final infection status, while the incidence also uses infection status at surveys during the course of the trial.

**Fig. 3:**
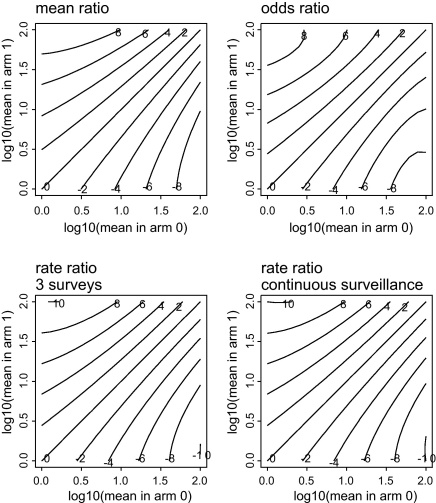
For small values of *k*, the ratio of means is not always the most powerful efficacy measure. In this example, *k* is proportional to the square root of the mean infection intensity, and equal to 0.5 when the mean is 100 (the highest value in the range considered). Other parameters are as in Fig. 2. In the current figure, the rate ratio is the most powerful efficacy measure. To see this, note that, for example, the rate ratio efficacy measures reach *Z* values of 10 for some combinations of means, while the other efficacy measures do not.

Similar patterns to those of Fig. 2 were seen for the other values of fixed *k* considered (0.1, 0.2 and 1, not shown). However, when *k* is allowed to vary with the mean, then the ratio of means is not always the most powerful effect measure. Such an example is shown in Fig. 3. Here, the incidence rate ratio is the most powerful, although the differences between the four effect measures are small.

### 3.2 Mass effects of trial interventions

We use the model developed in Appendix B to examine the reduction in force of infection which is caused by clearance of infections among participants at the start of the trial, and by the efficacy of the vaccine. Table 1 shows the default parameter values. For this model we assume that *k* is related to the mean. If, instead, *k* is assumed to be fixed and equal in the two arms, then, for large means, the value of the *Z* statistic depends on the means largely via the numerator, with the denominator being dominated by the values of *k* (see the equation for *Z*_*mean*_ in Appendix A). This implies that the power is effectively the same for a given ratio of means, irrespective of their magnitudes, which could well be misleading given the empirical evidence against assuming a fixed *k*.

**Fig. 4:**
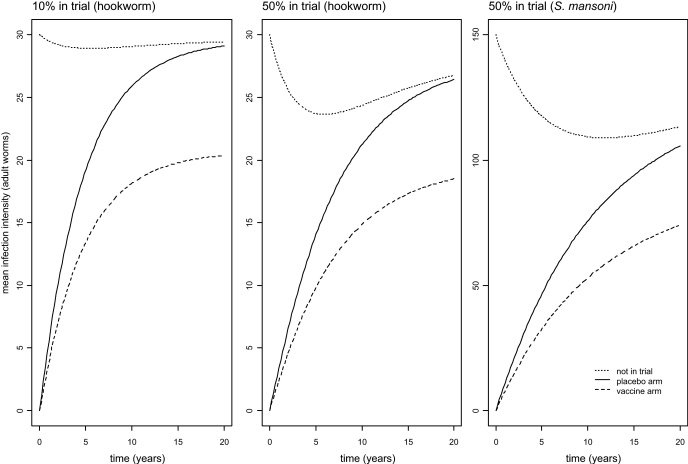
Examples of model predictions of the change in mean parasite intensity over time. People in the trial, whether in the vaccine or placebo arm, are assumed to have any initial infections cleared at baseline.

Three examples of the model output are shown in Fig. 4: the first two panels represent hookworm, and the rightmost one *S. mansoni* (see parameters in Table 1).

In each case, as expected, the new equilibrium mean is lower than the pre-trial equilibrium for unvaccinated people (whether in the control arm or outside the trial), because their force of infection from neighbouring participants is reduced. This effect is small when 10% of the study area's population are included (left hand panel), but appreciable when 50% are included (middle panel). In each panel, the new equilibrium in the vaccine arm is lower still, because the vaccine's efficacy is assumed to last indefinitely. The timescale for rebound to their respective new equilibria is similar in these two panels. By contrast, the new equilibrium level is reached more slowly in the case of *S. mansoni* (right hand panel). This is because the basic reproduction number (*R*_0_) is lower and the adult worm lifespan is higher (Table 1), and it is the ratio of these two quantities which controls the rate of spread of infection [31]. Hence values of these two parameters are likely to be important in planning vaccine trials, especially if baseline estimates of reinfection rates are not available.

**Fig. 5:**
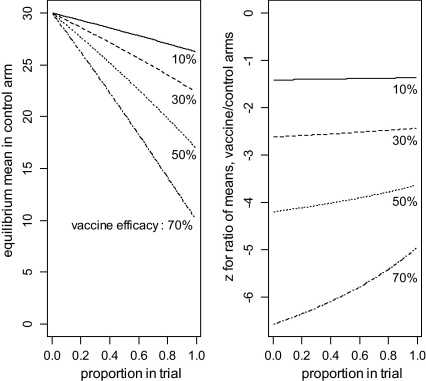
The effect of vaccine efficacy and proportion of population in the trial on (a) the post-trial equilibrium mean in the control arm (left panel) and (b) *Z* statistic for the ratio of mean parasite intensity in vaccine/control arms (right panel).

Fig. 5 shows that, if a greater proportion of people are in the trial, or the vaccine efficacy is greater, then (a) the new equilibrium mean is lower, and (b) the *Z* statistic is closer to the null value (i.e. less statistically significant). Moreover, we can see from right hand panel of Fig. 4 that the new equilibrium may not be approached for several years, quite possibly longer than is feasible for a trial. In such circumstances, the *Z* values in the right hand panel of Fig. 5 would over-estimate the statistical power of the trial.

## 4 Discussion

The first objective of the present work was to investigate the statistical properties of the between-arm ratio of mean parasite intensity at the end of a trial as an effect measure for vaccine trials, a suggestion we have made elsewhere [24]. The results show that, for the range of parameters considered, and under the assumptions made, the ratio of means is often much more powerful than the alternatives considered. However, there are also situations in which it is not the most powerful but, in those cases, the loss of power relative to other effect measures is small.

The negative binomial distribution, which we assumed for parasite counts, has proven to be a useful tool for analysing intensity of human intestinal helminth infections [21,22,29,32–36]. Nevertheless, a better fit to some datasets may be obtained by more sophisticated distributions such as the zero-inflated negative binomial [37,38]. As its name suggests, this is an elaboration of the negative binomial distribution, allowing for an increased proportion of parasite-negative individuals. The effect on the conclusions of the current work would depend on the extent of the zero inflation, which is likely to be rather imponderable at the trial planning stage. Although the current approach could be extended to other distributions, the two parameters of the standard negative binomial allow trialists to make assumptions for the mean and variance, and this is likely to be sufficient in many situations.

It is surprising that fitting a negative binomial distribution is not the most powerful analysis when this distribution is, by assumption, the one followed by the data. The fact that the ratio of means may be less powerful than the incidence rate ratio is explicable by the fact that the latter involves repeated measurements throughout the trial, while the former is based on just one final measurement. However, the ratio of means is also sometimes less powerful than the odds ratio (Fig. 3), despite the fact that it too is based only on a single final measurement. Our interpretation is that the negative binomial involves the estimation of two parameters, rather than one for the binomial and that estimation of *k*, when it has a small value, leaves less information for estimation of the means than is available for the odds ratio after dichotomizing the infection intensity data. However, in such cases the differences between the effect measures are small (Fig. 3).

It is striking that the benefit, if any, of the ratio of means depends crucially on the values assumed for the negative binomial *k* parameter. The simplest assumption, which we have made previously [24], is to assume that *k* is fixed and, in particular, that it is equal in the two arms of the trial. In practice, however, *k* is often found to increase with the mean [39]. Incorporating such patterns may reduce the statistical benefit of the ratio of means over the other effect measures. Moreover, if *k* is assumed fixed then, for large mean values, the required sample size would depend largely on the ratio of means and hardly at all on their individual magnitudes, which would be misleading when the assumption fails. In summary, values for *k* should be chosen carefully because the resulting sample sizes could vary greatly depending on the assumption made. Ideally, baseline studies would estimate mean and *k* in the vaccine study site, as well as the relation between these parameters across geographically defined subregions.

The current methods could be extended quite easily to look at the proportion over a given non-zero value, e.g. the thresholds used by the World Health Organization to define moderate or heavy infection intensity [40]. However, some other potential endpoints would require altogether different approaches. In particular, we did not consider the median infection intensity, largely because the majority of trial participants in each arm may well remain uninfected, in which case their median infection intensities would be zero. Nor did we consider the Williams mean [41], which is calculated by adding one to the parasite counts or intensities, then calculating the geometric mean, then subtracting one. This measure, sometimes loosely called the geometric mean [42], is dependent on the measurement scale [43] and, in the presence of a large proportion of zeros, has the additional problem that the desired normal (Gaussian) distribution [44] will not be obtained. Statistical convenience aside, the mean parasite intensity is more biologically relevant than these other intensity measures in that it is proportional to the total number of parasites present in the population. For example, in the case of hookworm, the mean infection intensity is proportional to the total number of eggs being excreted, which in turn is likely to be closely related to transmission intensity.

It would be possible to obtain an algebraic expression for the differences in *Z* statistics between the candidate effect measures, rather than calculating them numerically and making assessments from graphs. However, although the equations in Appendix A are not mathematically sophisticated, they are rather long, especially bearing in mind that each occurrence of *p* or *λ* is a function of *μ* and *k*, which are the starting points of the analysis. Hence it seems unlikely that pursuing the difference in *Z* statistics algebraically would be more informative than the graphical approach used here.

Basing the analysis on larger stool sample volumes (or more replicate samples taken per person at each examination round) will increase the mean (but not *k*, see Appendix C) and so will increase the power, since the denominator of *Z*_*mean*_ is reduced (Appendix A). The extent of this benefit will depend on the other parameters in the equation. Similarly, the stool sample volumes used by alternative parasite counting methods may influence the choice between them, e.g. Kato-Katz or McMaster for fecal egg counts. For some species, there may also be a choice of counting different forms of the parasite, e.g. adult worms or eggs, and these will, in general, have differing statistical powers depending on their distributions.

The second objective of the current work was to judge the extent to which population-level effects of trial activities – clearing baseline infections and inoculating some people with a vaccine hoped to be efficacious – are likely to reduce the force of infection and hence the statistical power of the trial. The results from our model suggest that these effects are likely to be unimportant for vaccines with low to middling efficacy (say, up to 50%). The magnitude of the test statistic decreases as a greater proportion of people are included in the trial, but the reduction in power is large only for high efficacies (Fig. 5). One additional conclusion from the model is a reminder that the dynamics of infection may be rather slow, and, for some species, trials may well be too short for a new equilibrium situation to be reached (Fig. 4). The return will be quicker for larger values of the basic reproduction number (*R*_0_), shorter-lived parasites [31], and trial designs in which a smaller proportion of residents receive an effective vaccine.

This model is a simple extension of an existing one [22]. It was necessary to extend the model to represent the various groups in a vaccine trial, but some features of the previous model were omitted. In particular, we did not consider the fact that some species are dioecious and so may fail to reproduce due to lack of a mate in their human host. This may become an important constraint at low infection intensities. Nor did we allow for the time taken to develop to sexual maturity in the human host. For hookworm, this period is approximately 6–8 weeks [45] which is likely to be short compared to the time needed to return to equilibrium, so this factor is likely to be relatively unimportant. We also assumed random mixing – i.e. that an uninfected person is equally likely to acquire infection from any of the infected people in the population – which is a simplification, possibly an over-simplification in some cases.

The vaccine efficacy was expressed as a constant factor (*ψ*) applied to the rate of acquisition of infection, in terms of numbers of adult worms. We did not look at other modes of action, e.g. a proportion (*ψ*) of the vaccine arm being completely protected and the remainder receiving no protection [46]. The present work could be modified to divide the vaccine arm into two groups, although it seems unlikely that the mass effects would be changed greatly. Another limitation is the assumption of lack of waning of vaccine efficacy. Rapid waning would clearly have a major impact, especially in the vaccine arm, although quantifying this would, given current knowledge, be rather speculative.

The model is simpler than the microsimulation approach used in a related study [47] which focussed on the effects of differential vaccine efficacy in groups with different infection intensity. The earlier study estimated vaccine effects over time on both infection and morbidity, while the current paper concentrates on how most efficiently to measure vaccine effects on infection. We have assumed that the negative binomial dispersion parameter (*k*) varies with the mean, rather than staying constant. The former assumption is better supported by available data and is likely to crucially affect statistical power.

We have only considered a very simple trial design, with participants individually randomized between vaccine and control arms. Mass effects could be reduced by using other designs, in particular cluster-randomization [48]. However, our results suggest that, if only a relatively small proportion of the study population receive the vaccine, then such effects are, in any case, likely to be relatively unimportant. If the study population is children then such effects are likely to be even less because, although age does not appear explicitly in the model presented here, children will tend to have infections of lower intensity.

We conclude that mean parasite intensity is a feasible endpoint for later phase vaccine trials. In addition, although the trial interventions will reduce the force of infection in the study area, the resulting loss of statistical power is unlikely to be important unless there is a combination of high vaccine efficacy and a high proportion of population enrolment. Finally, the magnitude of some results depended greatly on whether the negative binomial *k* parameter was assumed fixed or allowed to vary with the mean, and such assumptions should be considered carefully when planning trials.

## Figures and Tables

**Table 1:** Default parameter values for the model of mass effects in vaccine trials.

Parameter		Parasite	Value	Sources
*R*_0_	Basic reproduction number	Hookworm	4	Ye et al. [54]. Other reported values are lower, e.g. 2–3 by Anderson and May in their Table 16.3 [22], and approximately 2 by Bradley et al. [33], but this higher value gives better agreement with our unpublished data
		*S. mansoni*	2.5	Woolhouse [55]
*ω*	Death rate in humans		0.025/year	
*ω*_1_	Death rate of adult worms	Hookworm	0.2/year	Loukas and Prociv [56]
		*S. mansoni*	0.125/year	To illustrate possible differences with hookworm we have chosen a value which corresponds to a lifespan of 8 years, towards the upper end of the following published estimates: 6–10.5 years by Fulford [57], 4–9, ≥8 and ≥16 estimated by Vermund et al. in different cohorts [58], and 3–5 years by Goddard and Jordan [59] and Anderson and May [22, p. 438]
	Grams of faeces per person per day		150	Towards the lower end of developed country estimates [60,61]
	Eggs per female worm per day	Hookworm	10,000	Bethony et al. [1]
		*S. mansoni*	300	LoVerde et al. [62], Anderson and May [22]
μ^*^	Equilibrium mean number of adult worms per person	Hookworm	30	Calculated from mean of about 1000 eggs per gram (epg) of faeces in Americaninhas (Brazil) [29], times 2 to include male worms, times grams of human faeces per day (above), divided by eggs per female worm per day (above)
		*S. mansoni*	150	As above but using 150 epg [29]
*k*	Dispersion parameter for negative binomial distribution of adult worms per person, at the equilibrium mean (μ^*^)	Hookworm	0.35	From Bradley et al. [33]. In Americaninhas (Brazil) some of the present authors found *k* ≈ 0.1 for egg counts [29] but the distribution of adult worms was not estimated, As the mean varies, *k* is assumed to be proportional to its square root
		*S. mansoni*	0.35	As previous row. The *k* for *S. mansoni* egg counts in the study area was approximately 0.2 [29]
*d*	Strength of density dependence	Hookworm	0.9791	Chosen to obtain stated value of μ^*^: *d* = 1 − (*k*/μ^*^)(*R*_0_^1/(*k*+1)^ − 1)
		*S. mansoni*	0.9977	As above
*ψ*	Factor by which vaccine reduces rate of acquisition of adult worms		0.7 (30% efficacy)	

## Box Worked example

We revisit the example given by Brooker et al. [24]. In the control arm of a trial, the final mean number of hookworm eggs counted per person is assumed to be *μ*_0_ = 72, with the negative binomial dispersion parameter being *k*_0_ = 0.33. The mean in the vaccine arm is assumed to be 30% less: *μ*_1_ = 72×(1 − 0.3) = 50.4. Assuming that *k* is proportional to the square root of the mean, the vaccine arm has $k_{1}=k_{0}\sqrt{\mu 1/\mu 0}\approx 0.28$.

The egg-positive prevalences in the control and vaccine arms are then 83% and 76%, respectively (using the relevant equation in Appendix A), and the corresponding incidence rates are 1.78 and 1.44. These rates are expressed with trial length as the unit. So if the trial takes 1 year to reach the given infection intensities, then the rates are per year. If the trial takes two years to reach the same infection intensities, then the rates are 0.89/year and 0.72/year, respectively.

Comparing effect measures, the *Z* value for ratio of means – i.e. the log ratio of means divided by its standard error – with 100 people per arm is *Z*_*mean*_ = 1.38. This is larger than the corresponding value for odds ratio (*Z*_*OR*_ = 1.20) and slightly larger than those for rate ratio based on 3 surveys (*Z*_*RR*_ = 1.32) or continuous surveillance (*Z*_*RR*_ = 1.34). Finally, the sample size based on ratio of means, for 90% power and 5% two-sided significance level, is 523 per arm, compared to 505 per arm with *k* = 0.33 for both arms [24].

## Appendix A Algebraic relations between the candidate trial endpoints and effect measures

The egg counts are assumed to follow a negative binomial distribution with parameters *μ* (the mean) and *k*. This distribution applies to the number of parasites seen, before conversion to a standard amount such as eggs per gram. We denote the mean egg counts, at the end of the trial follow-up, in the intervention and control arms of the trial by *μ*_1_ and *μ*_0_ respectively. Similarly, the second parameter of the negative binomial distribution is denoted by *k*_1_ and *k*_0_ respectively in the two arms. In some cases we assume *k*_*1*_ = *k*_0_. Each arm is assumed to have data on *n* participants.

To compare the mean parasite intensity between the intervention and control arms, we consider the test statistic *Z*_*mean*_, which is the difference in log-means divided by the standard error of this difference [24]:

$$Z_{\text{mean}}=\frac{\log(\mu_{1})-\log(\mu_{0})}{\sqrt{\text{var}(\log(\mu_{1})-\log(\mu_{0}))}}=\frac{\log(\mu_{1})-\log(\mu_{0})}{\sqrt{\left(1/n)((1/\mu_{1})+(1/\mu_{0})+(1/k_{1})+(1/k_{0})\right)}}$$

The prevalence of infection (*p*) is related to the negative binomial parameters as follows [22]:

$$\text{Prevalence}=\text{Probability}\;(\text{positive})=p(\mu ,k)=1-{\left(\frac{k}{\mu +k}\right)}^{k}$$

Hence from the means in the two arms (*μ*_0_ and *μ*_1_), and *k*, we can derive the prevalences (*p*_0_ and *p*_1_), and so the odds of being positive, in the two arms at the end of the trial. From this we derive the test statistic for the odds ratio between the arms *Z*_*OR*_, which is the ratio of the difference in log-odds divided by the standard error of this difference [49]:

$$Z_{or}=\frac{\log\left(\frac{p_{1}}{1-p_{1}}\right)-\log\left(\frac{p_{0}}{1-p_{0}}\right)}{\sqrt{\text{var}\left(\log\left(\frac{p_{1}}{1-p_{1}}\right)-\log\left(\frac{p_{0}}{1-p_{0}}\right)\right)}}=\frac{\log\left(\frac{p_{1}}{1-p_{1}}\right)-\log\left(\frac{p_{0}}{1-p_{0}}\right)}{\sqrt{\frac{1}{n}\left(\frac{1}{p_{1\left(1-p_{1}\right)}}+\frac{1}{p_{0\left(1-p_{0}\right)}}\right)}}$$

We can relate the time to first infection to the other endpoints via the rate (*λ*) at which trial participants become positive. If the trial lasts time *T*, then the proportion positive at the end of the trial (*p*) is related to *λ*, *μ* and *k* as follows:

$$\lambda =-\frac{\log(1-p)}{T}=k\;\frac{\log(k/(\mu +k))}{T}$$

For many parasites, the time of infection is not marked by specific symptoms and so will not generally be observed exactly. Rather, the infection will be detected at the next follow-up examination. This reduces the precision of the rate estimation. If we assume that there are *m* examinations evenly spaced throughout the trial, then the sampling error can be quantified as a decreasing function of *m*, as can the Fisher information *I*_*λ*_ of the incidence rate *λ* [30]. The Fisher information for the natural logarithm of the rate *I*_log(*λ*)_ is *I*_*λ*_ divided by the square of the differential of the logarithmic function, so *I*_log(*λ*)_ = *λ*^2^*I*_*λ*_. Hence we obtain the *Z* statistic for the rate ratio (*λ*_1_/*λ*_0_):

$$Z_{RR}=\frac{\log(\lambda_{1})-\log(\lambda_{0})}{(T/m)\sqrt{\left(1/n)((((e^{\lambda_{1}T/m}-1)(1-e^{-\lambda_{1}T/m}))/(\lambda_{1}^{2}(1-e^{-\lambda_{1}T})))+(((e^{\lambda_{0}T/m}-1)(1-e^{-\lambda_{0}T/m}))/(\lambda_{0}^{2}(1-e^{-\lambda_{0}T})))\right)}}$$

We assume *T* = 1 so that the incidence rates are per study duration. Since *p*_0_, *p*_1_,*λ*_0_ and *λ*_1_ can all be expressed in terms of *μ* and *k*, we can now directly compare the magnitudes of the test statistics of the three effect measures: the ratio of means, the odds ratio, and the incidence rate ratio.

## Appendix B Mathematical model of the impact of trial interventions on the force of infection

We use a model of Anderson and May [22] for the mean infection intensity *μ*, which now refers to the number of adult worms per person (rather than eggs counted per sample):

(A.1) $$\frac{d\mu }{dt}=(\omega +\omega_{1})(R_{0}f(\mu )-1)\mu$$

In this equation, *R*_0_ is the basic reproduction number, *ω* and *ω*_1_ are the death rates of humans and adult worms, respectively, and the following term

$$f(\mu )={\left(1+\frac{\mu (1-d)}{k}\right)}^{-k-1}$$

represents density dependence via a parameter *d*. More specifically, *d* is the ratio by which per capita egg output decreases per extra adult. We impose density dependence (*d* ≠ 1) because otherwise the model has no stable non-zero equilibrium.

We divide the population of the trial study area into three groups. A proportion *ϕ* are in the trial, half each in the active vaccine and control arms. People in both arms are assumed to have their infections cleared at the start of the trial. The third group, comprising a proportion 1 − *ϕ*, are not in the trial and receive no intervention. The rates at which the three groups acquire infection from each other are assumed to be proportional to the fraction of the population which they comprise. Finally, the vaccine is assumed to reduce acquisition of infection, in terms of numbers of adult worms, by a factor *ψ*. So we can write the following equation for the mean *μ*_1_ in vaccine arm:

$$\frac{d\mu_{1}}{dt}=(\omega +\omega_{1})\left(R_{0}\psi \left(\frac{\phi }{2}f(\mu_{1})\mu_{1}+\frac{\phi }{2}f(\mu_{0})\mu_{0}+(1-\phi )f(\mu_{2})\mu_{2}\right)-\mu_{1}\right)$$

Here, *μ*_0_ and *μ*_2_ are the means in the control arm and the non-trial groups respectively. The equation for the placebo has a similar form but without *ψ*. The non-trial group has the same equation as the placebo arm, but differs in that its initial value is the equilibrium value *μ*^*^ rather than 0.

## Appendix C Effect of sample volume on negative binomial parameters

This paper generally assumes that over time, or between arms of the trial, higher values of mean egg count are associated with higher values of the negative binomial dispersion parameter *k*. Such an association does not apply, however, when the increased mean results simply from counting a larger sample volume per person.

It is well known that the sum of *n* independent negative binomial random variables, each with parameters *μ*_0_ and *k*_0_, itself follows a negative binomial distribution with parameters *n* × *μ*_0_ and *n* × *k*_0_ [50]. Hence one might think that, for example, doubling the sample volume read per sample – e.g. by doubling the number of fecal slides – would double both *μ* and *k*. This is not the case, however, because the additional sample is not independent of the original one, contrary to the premise of the calculation.

The negative binomial distribution can be derived as a gamma mixture of Poissons [51]: within each person the stool (or blood, etc.) is sampled uniformly (a Poisson process with fixed mean), but the between-person variation is modelled by a gamma distribution. Equivalently, each person's Poisson variate has a mean equal to the population mean, multiplied a ‘random effect’ with distribution gamma(*k*)/*k*, and hence mean 1 and variance 1/*k* [52]. Increasing the sample volume increases the mean but not the person's random effect: it's not possible to reduce between-person heterogeneity by processing larger samples.

This can be verified empirically by simulating a given number of negative binomially distributed parasite counts, then halving each sample by allocating each parasite at random to one of two subsamples. The estimated *k* parameter for each of the subsamples is the same (within sampling error) as that for the larger sample. As Morton and Baird express this, the dispersion parameter is ‘invariant under random thinning’ [53].
